# Accelerating Spaceborne SAR Imaging Using Multiple CPU/GPU Deep Collaborative Computing

**DOI:** 10.3390/s16040494

**Published:** 2016-04-07

**Authors:** Fan Zhang, Guojun Li, Wei Li, Wei Hu, Yuxin Hu

**Affiliations:** 1 College of Information Science and Technology, Beijing University of Chemical Technology, Beijing 100029, China; 2013200783@mail.buct.edu.cn (G.L.); liw@mail.buct.edu.cn (W.L.); huwei@mail.buct.edu.cn (W.H.); 2 Institute of Electronics, Chinese Academy of Sciences, Beijing 100190, China; yxhu@mail.ie.ac.cn

**Keywords:** synthetic aperture radar (SAR), advanced vector extensions (AVX), graphics processing unit (GPU), imaging algorithm, collaborative computing

## Abstract

With the development of synthetic aperture radar (SAR) technologies in recent years, the huge amount of remote sensing data brings challenges for real-time imaging processing. Therefore, high performance computing (HPC) methods have been presented to accelerate SAR imaging, especially the GPU based methods. In the classical GPU based imaging algorithm, GPU is employed to accelerate image processing by massive parallel computing, and CPU is only used to perform the auxiliary work such as data input/output (IO). However, the computing capability of CPU is ignored and underestimated. In this work, a new deep collaborative SAR imaging method based on multiple CPU/GPU is proposed to achieve real-time SAR imaging. Through the proposed tasks partitioning and scheduling strategy, the whole image can be generated with deep collaborative multiple CPU/GPU computing. In the part of CPU parallel imaging, the advanced vector extension (AVX) method is firstly introduced into the multi-core CPU parallel method for higher efficiency. As for the GPU parallel imaging, not only the bottlenecks of memory limitation and frequent data transferring are broken, but also kinds of optimized strategies are applied, such as streaming, parallel pipeline and so on. Experimental results demonstrate that the deep CPU/GPU collaborative imaging method enhances the efficiency of SAR imaging on single-core CPU by 270 times and realizes the real-time imaging in that the imaging rate outperforms the raw data generation rate.

## 1. Introduction

SAR is a kind of active-observation system to the Earth, which can be installed in the aircraft, satellite and spacecraft flying platform [1]. With the imaging characteristics of day-and-night, all-weather, and surface-penetration, the SAR system has been widely applied to the disaster monitoring, resource exploration, environmental monitoring, marine monitoring, and crop yield estimation [2,3]. Therefore, the development and research on SAR attract more and more attention of the countries around the world, such as system design, raw data simulation, imaging algorithm, information extraction and so on. Among them, the raw data simulation and imaging algorithm not only verify the availability of SAR system, but also bridge the SAR system design and remote sensing applications.

To quantitatively support the SAR system design of multiple working modes, to help mission planning, and to test processing algorithms, a SAR raw signal simulator is required to generate raw data especially when real raw data are not available yet [4]. Normally, there are two levels of raw data simulators, respectively are point target simulator and extended scene simulator. The point target simulator consisting of one or a few point scatters, is usually employed to test the focusing capability of imaging algorithms. Further, the extended scene SAR raw signal simulators would be suitable to quantify the performances of imaging algorithm at variance of the scene and SAR parameters both in terms of achieved SAR geometrical resolutions and radiometrical resolutions. Accordingly, the extended scene raw signal simulators of multiple modes and observed scenes were gaining an increasingly wide interest, and extensively studied, such as strip map SAR [5,6,7], spotlight SAR [8], hybrid stripmap/spotlight SAR [4] and so on. Using the extended scene simulators, the effects of imaging accuracy could be emphasized when complex targets are considered, so that they can vary for different kinds of observed scenes and hence for different applications, such as ocean [9] and urban [10]. Especially, the new generation of spaceborne/airborne SAR sensors are developed towards high resolution, which brings various objects and rich features in the SAR images. It would be highly desirable that an extended scene SAR simulator is planned in order to identify the optimum SAR system configuration, operational mode and imaging algorithms with reference to the considered application [10].

In recent years, Chirp Scaling (CS) algorithm has become the mainstream in SAR imaging field [11]. One of the important contributions is that the range cell migration correction (RCMC) [12,13] interpolation operation is avoided compared to Range-Doppler (RD) algorithm, thus the amount of computation is reduced about 1.4 times [14]. Besides, CS algorithm can significantly improve the performance of the SAR imaging because of the accurate processing in the squint mode [2,15,16]. On the other hand, Back-Projection (BP) algorithm is theoretically capable of precise imaging processing for various mode, but is seldom employed in practical applications due to low imaging efficiency. So, efficiency is the most important motivation of SAR imaging algorithm development. Due to the advantage of high efficiency, CS and its improved algorithms are still employed for spaceborne SAR imaging of various modes, including Strip mode [17], Spotlight mode [18], Scan mode [19], Sliding Spotlight mode and TOPS mode [20]. With the development of SAR technologies, there is a higher demand for the resolution and swath of SAR images, which brings a huge amount of imaging calculation. Particularly, the imaging algorithm is adjusted many times to adapt to the flight conditions in the initial stage of system launching. Therefore, the fast and even real-time imaging processing is strongly required in the ground processing system.

Due to the limited efficiency improvement from the perspective of signal processing, the most straightforward idea for accelerating the SAR imaging is the parallel algorithm design based on high performance computing (HPC) methods. Primordially, there are two kinds of HPC methods applied to SAR imaging processing in general. One is the global shared memory system, and the other is the cluster system consisting of several independent nodes [21]. Gradually, there are some researchers transplant SAR imaging algorithms to special DSP [22] and FPGA [23] processors while existing various limitations of universality. There are also several general parallel solutions implemented on CPU based on OpenMP and MPI [24], but they are still not at the level of real-time processing.

For now, due to the driving of the market of graphic processing, Graphic Processing Unit (GPU) has been developed as a high performance processing platform with characteristics of high parallelism, multi-threading, many-cores, huge bandwidth capacity and with hundreds of computing cells [1,25]. The birth of Compute Unified Device Architecture (CUDA) promotes GPU to be widely used in the high performance computing field. With the help of CUDA technology, those traditional code implements large amounts of floating point computation with CPU can be easily ported to GPU to realize massive parallelism. The existing results of research indicate that GPU based method can improve the efficiency of SAR imaging by dozens of times [3,11,26,27,28,29,30], which outperforms the traditional HPC methods. However, there are still some further optimizations for the existing methods to accelerate SAR imaging processing. For example, the issue of data transferring is not solved in Song’s method [11]. In addition, just 2 Tesla C2050 cards are used in current papers [11,27]. Though the GPU based method can process huge amount of raw data and boost the computing efficiency many times, they still cannot meet the practical requirement for fast or even real-time SAR imaging.

In addition, the computing power of CPU is underestimated or even ignored in current GPU methods. Typically, CPU only takes on tasks of logical processing, controlling and data input/output (IO) [3,11]. Then other researchers further improve the GPU method by exploiting more computing power of CPU, which is realized by the multi-core parallelism [27]. The efficiency of multi-core CPU and GPU are in the different level, which makes the contribution of CPU computing unimpressive. From the view of computationally intensive imaging processing, CPU is still a co-processor of GPU rather than a real computing processor. Therefore, there are three major limiting factors in current GPU-based SAR imaging algorithms, which are described as follows:

- The first one is the device memory of GPU, which limits the size of processed raw data. To break the limitation, more complicated processing should be applied to the original imaging algorithm, such as sub-aperture processing.
- The second one is the underestimation of CPU parallel computing, which reduces the parallel efficiency of the imaging computation.
- The third one is the lack of substantive collaborative computing solution, which ensures the scalability for heterogeneous parallel computing resources and meets the requirement of real-time imaging.

The emergence of Single Instruction Multiple Data (SIMD) instructions, namely advanced vector extensions (AVX), provides a chance to deeply improve the parallel computing capability of CPU. The combination of AVX and multi-core parallel can make the computing capability of CPU comparable with GPU. Our previous works [31,32,33] have a preliminary discussion on this, and proved that CPU had competitive computing power. In terms of that, CPU no longer just takes on some auxiliary tasks of SAR imaging processing, but can really become more involved in the computing works of imaging processing. The computing resources of CPU should be taken into account when designing a parallel strategy to realize the real-time CS algorithm. Thus, a deep multiple CPU/GPU collaborative imaging method, which makes multi-core SIMD CPU and many-core GPU work together to speed up SAR imaging processing, is proposed. In each step of the imaging process, computing task is divided and dispatched to CPU and GPU separately, and then achieving the substantial collaborative imaging considering multiple heterogeneous processor. The key point of CPU/GPU collaborative computing is that we need to schedule the task reasonable to make them fulfill the tasks synchronously. So, the fine-grained partitioning method is employed to balance the workload of CPU and GPU [34,35,36]. Compared to the existing research, we mainly make the following contributions:

- A bi-direction partitioning strategy is proposed to solve the contradiction between big raw data and limited GPU memory. It not only provides support for the multiple devices based parallel imaging, but also avoids the complex sub-aperture processing [27], which is employed by existing multi-GPU based CS imaging to solve the aforementioned contradiction;
- A vector processing based multi-core CPU parallel imaging method is presented. Through the fine-grained vectorization parallel and coarse-grained multi-core parallel, the computing power of CPU is strengthened, and the gap between CPU and GPU computing is greatly narrowed;
- A deep multiple CPU/GPU collaborative computing method is presented to accelerate SAR imaging processing. CPU is no longer the auxiliary processor of GPU, but the main computing processor taking a considerable part of the computing task. Meanwhile, the multi-GPU based CS imaging algorithm is further optimized by streaming, parallel pipeline and other strategies.

The rest of this paper is organized as follows. We introduce the background of CS algorithm and parallel models of CPU and GPU in Section 2. The multiple CPU/GPU collaborative imaging method and its details of the parallel optimizing strategies are described in Section 3. Then, Section 4 provides the experimental results and the corresponding analysis. Finally, conclusions are drawn in Section 5.

## 2. Methodology

### 2.1. CS Algorithm

CS algorithm has become the most popular SAR data processing algorithm. The essence of this algorithm is that the calculation of interpolation in the processing of range cell migration correction (RCMC) is avoided, which would consume much computing resources [2]. For the center frequency and bandwidth that may be changed by increasing scaling or translation of frequency, CS algorithm realizes RCMC by two steps. Firstly, correcting the range cell migration curve of targets at different locations in the range by compensating the spatial variation of range cell migration in Range-Doppler domain. The Chirp Scaling quadratic phase function used to adjust the characteristics of space-varying in range can be expressed as following,

(1) $$H_{1}\left(\hat{t},f_{a};r_{0}\right)=\exp\left[-j\pi k\left(f_{a};r\right)a\left(f_{a}\right){\left(\hat{t}-\frac{2R(f_{a};r_{0})}{c}\right)}^{2}\right]$$

where $\hat{t}$ is time variable in range direction, $f_{a}$ is azimuth frequency, $r_{0}$ is the reference distance, $k(f_{a};r)$ is the modulating frequency in range direction, and $R(f_{a};r_{0})$ is the range migration curve of the reference distance. Note that $a(f_{a})$ can be described as,

(2) $$a\left(f_{a}\right)=\frac{1}{\sqrt{1-{\left(\frac{\lambda f_{a}}{2v}\right)}^{2}}}-1$$

After Chirp Scaling, Fourier transform is implemented on range part of signal to make it into two-dimensional frequency domain, which can be expressed as $f_{r}$, $f_{a}$ domain, where *f* means frequency, *r* and *a* represent the range and azimuth direction, respectively.

Then the remaining range cell migration is corrected precisely by multiplying a reference function,

(3) $$H_{2}\left(f_{r},f_{a};r_{0}\right)=\exp\left[-j\pi \frac{1}{k\left(f,r_{0}\right)\left[1+a\left(f_{a}\right)\right]}f_{r}^{2}\right]\;\;\cdot \exp\left[\frac{4\pi r_{0}a\left(f_{a}\right)}{c}f_{r}\right]$$

At last, the focusing of azimuth direction can be realized by multiplying by a matching function,

(4) $$H_{3}\left(\hat{t},f_{a};r\right)=\;\;\;\cdot \exp\left[j\frac{4\pi }{\lambda }r\sqrt{1-{\left(\frac{\lambda f_{a}}{2v_{0}}\right)}^{2}}\right]$$

The CS algorithm achieves the two dimensional focusing through a series of phase processing. Furthermore, the issue that Secondary Range Compression (SRC) always depends on azimuth frequency which is taken into account in this algorithm. Therefore, the effect of secondary range compression can outperform RD (Range Doppler) algorithm. Meanwhile, its processing efficiency is superior to RD algorithm [2]. As a consequence, CS algorithm is more efficient and precise in that there are only FFT and multiplication operations in the imaging processing. It is suitable for signal processing of the space-borne strip map SAR data.

**Figure 1:**
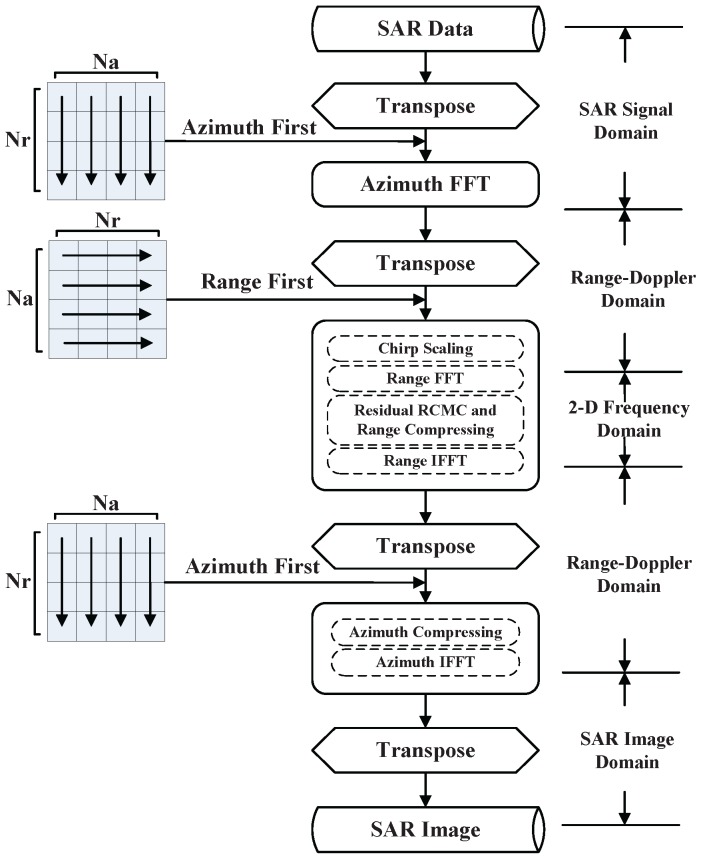
The chirp scaling algorithm diagram.

The flowchart of CS algorithm is illustrated in Figure 1. Firstly, the initial SAR raw data that arranged by range first should be transferred to the range doppler domain. Secondly, the chirp scaling operation is executed to make all the range migration curves same. Third, the range compressing and RCMC are carried out in 2-*D* frequency domain. Finally, the image is obtained by azimuth processing. Due to different processing directions, there should be four times of transpose operation through the overall CS algorithm. To better understand the following strategy of parallel optimization, the procedure of CS algorithm is divided into 3 parts by nodes of transpose operation, which represents FFT in azimuth, processing in range direction, and operations in azimuth, respectively. Due to the huge volume of SAR raw data, the fast imaging and even real-time imaging are strongly in demand in practical applications. Therefore, the fast imaging algorithm becomes a hot topic in SAR imaging domain.

### 2.2. GPU Based SAR Massive Parallel Imaging Algorithm

**Figure 2:**
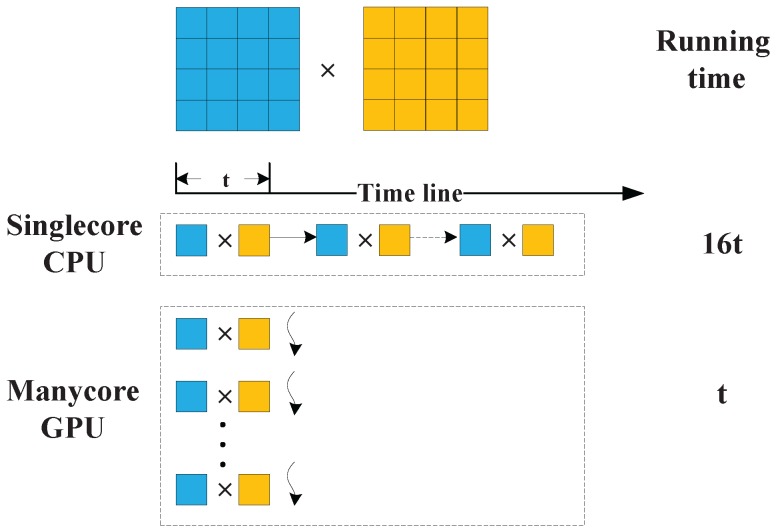
The comparison of phase multiplication between CPU and GPU.

GPU cards of NVIDIA have been developed as a high performance computing platform with high parallelism, many-cores and ultra high bandwidth, and proved to be high efficiency. It can be applied to operation platform for computing tasks which are floating and parallel computing based by using CUDA. CS algorithm is a regular computing method, which is easily designed to GPU based parallel algorithm. The CS algorithm mainly consists of phase multiplication, transpose and FFTs, which can be implemented on GPU with high performance. In the case of phase multiplication, the traditional single-core CPU method sequentially calculates the data item multiplication point by point, and the many-core GPU method concurrently calculates the multiplication in same time line, as illustrated in Figure 2. Similarly, the transpose and FFT operation are executed in parallel computing mode, which greatly accelerates the imaging process. As for the implementation of GPU based CS algorithm, it is more matured. Some of the operations can be implemented by calling the existing function libraries of CUDA, such as the case that the FFT operation calls the functions of CUFFT. The rest operations that cannot find any libraries to call can be implemented by writing corresponding kernels.

To further improve the efficiency of the GPU based CS algorithm, several optimizations can be applied. First, combining streams with asynchronous concurrent execution and reasonable partitioning of SAR data, the time consumption of memory copy between CPU and GPU can be hidden. Second, CUDA also provides opportunity for faster memory access by sharing memory, which can be applied into transpose of matrix. Third, any operations may lead to results that go against CUDA programming should be avoided. Similar to bank conflicts which may occur in using of shared memory, non-coalescing memory and non-page-locked memory access results in extra time consumption. Finally, multi-GPU technology should be introduced to speed up the algorithm further for the independence of raw data.

Compared with single GPU based CS algorithm, multi-GPU version is capable of breaking through the limitations of device memory. Namely, the multi-GPU version can process large SAR raw data with data partitioning and scheduling. However, there is a data transpose issue that is still not drastically solved in current multi-GPU imaging method. Due to two dimensional processing, several transpose operations are performed during the imaging stage. For huge data transpose operation, large memory space is the necessary condition, which cannot be met by the GPU memory. Therefore, the transpose operations are always implemented by CPU in the traditional multiple GPU imaging algorithm. Obviously, the GPUs are all awaiting while CPUs are doing the transpose operation, thus the parallel efficiency slows down. In the paper, we mainly focus on solving this issue and improve the parallelism of multi-GPU based CS imaging algorithm.

### 2.3. CPU Based SAR Imaging Processing with Multi-Core Vector Extension

As mentioned in the introduction, the method of further digging the computing power of CPU is the vector extension computing model, which can make CPU comparable with GPU in computing efficiency. In this case, the computing power of CPU can not be underestimated anymore. CPU is no longer just an auxiliary processor of GPU, but acts as a computing processor to take on a part of computing tasks.

It is undeniable that OpenMP is a powerful tool to realize parallel optimization of CS algorithm at a multi-core level. However, the technique is insufficient to satisfy the demand of real-time SAR imaging. To further enhance the parallel efficiency on CPU, more in-depth optimization with vector extension computing model is required. From the point of parallel granularity, multi-core CPU is coarse-grained parallelism, and the vector extension can be seen as a fine-grained strategy. Vector extension model can optimize program with data-level parallelism [37]. The SIMD CPUs extend the vector extension model with the introduction of the SSE and AVX [33]. According to the existing literature, one of the important trends in technologies and computing platforms is that vectors are playing an increasingly important role in both memory accesses and arithmetic operations [35].

**Figure 3:**
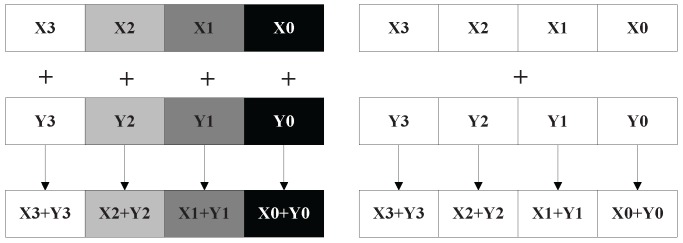
The operating model of SISD (**Left**) and SIMD (**Right**).

AVX vector length increases to 256 bits, which is longer than SSE of 128 bits. SSE/AVX allows vector extension computations to perform on the operands containing 4 or 8 pieces of packed single precision floating point data. The operands can be stored in memory or 128 bits or 256 bits registers further more. The main advantage of the vector extension model is capable of performing 4 or 8 pieces of single precision floating point data or 2 or 4 pieces of double precision floating point data with one parallel instruction. Theoretically, the application of vector extension model can improve 4 to 8 times at least. The models of SISD (Single Instruction Single Data) and SIMD are illustrated in Figure 3. For example, 4 pieces of single precision data can be added with one instruction in SSE. As for the traditional SISD CPU, one instruction can only add one piece of data. Due to the fact that CS imaging is a regular and continuous process, which is suitable for employing the vector extension computing model.

## 3. CPU/GPU Collaborative Computing Based SAR Imaging Processing

The existing fast SAR imaging processing methods on GPU always have some obvious problems that may limit the performance of accelerating. In paper [11], MingCong proposed to realize fast SAR image processing, which executed transpose operations on CPU, and then transferred data to GPU to finish the rest processing. There are frequent data transferring between GPU memory and CPU memory because the transposing operation is implemented only on CPU. Meanwhile, there exists waste of computing resources in that GPU is idle while executing transpose operation on CPU, and CPU is idle while doing multiplication and FFT on GPUs. This is not the most effective method to utilize the computing resources of both CPU and GPU.

Another typical GPU based imaging processing [28] is that the whole processing procedure is implemented on GPU, and CPU only takes on some workloads such as controlling and I/O. There are two disadvantages about this method. First, the volume of GPU memory is limited. With the size of SAR data increasing, GPU memory is not large enough for the whole chunk of SAR raw data. Second, CPU is always idle when GPU is computing, which is a waste of computing resources of CPU.

Considering the disadvantages of existing methods, a CPU/GPU collaborative computing based SAR imaging algorithm is proposed. Three obvious flaws of the exiting methods are avoided in the proposed method.

**Figure 4:**
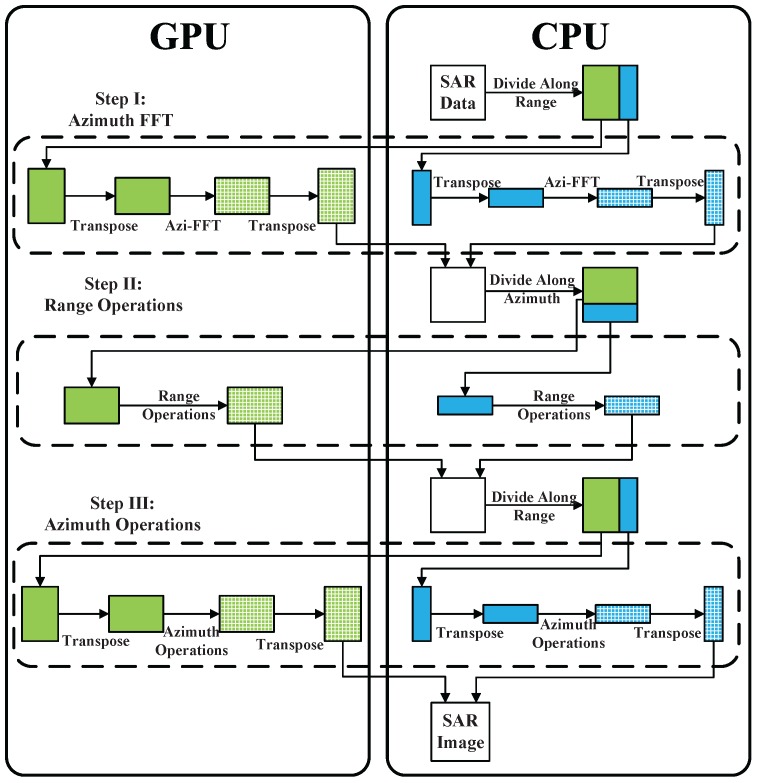
Bi-direction partitioning based multiple devices parallel imaging.

First, the limitation of GPU memory is broken through the proposed bi-direction partitioning strategy, through which transpose operation can be separately performed on both GPU and CPU. Accordingly, the frequent data transferring between CPU and GPU for transpose operation is avoided. Besides, the part of SAR data processed by GPU can be copied to GPU memory, followed by starting computing without waiting for data transpose on CPU. What’s more, the size of copying data is smaller, because there is only a part of data stays at CPU memory to be processed. A flowchart of the bi-direction partitioning based multiple devices parallel imaging is illustrated in Figure 4.

SAR raw data can be accessed on CPU at first, then it is partitioned into 2 parts. One of them is copied to GPU memory and processed by GPU, and the other is processed by CPU. Then, GPUs and CPUs can be controlled by multi-thread, so that they can work synchronously. Azimuth FFT operation is the first step of CS algorithm, including matrix transposing, azimuth FFT, and matrix transposing one more time, which are implemented on both GPU and CPU. After Step I, data is copied from GPU to CPU. SAR data is expected to be arranged by range first in the next step. As a consequence, the two parts of data should be collected together to prepare for the processing of Step II. Procedure of Step II and Step III can refer to Step I, which are similar. As shown in Figure 4, the green bigger rectangle block represents the work load assigned to GPU, and the blue smaller block is the work load assigned to CPU. Solid color means that data is unprocessed (not include transposing), and the blocks with mesh mean that this block of data is processed.

Second, CPU also plays an important role of SAR imaging. The vectorization and multi-core parallel based CS algorithm is presented so that all the imaging steps are executed on multi-CPUs. The only difference between GPU and CPU is the task load because of their different computing capability.

**Figure 5:**
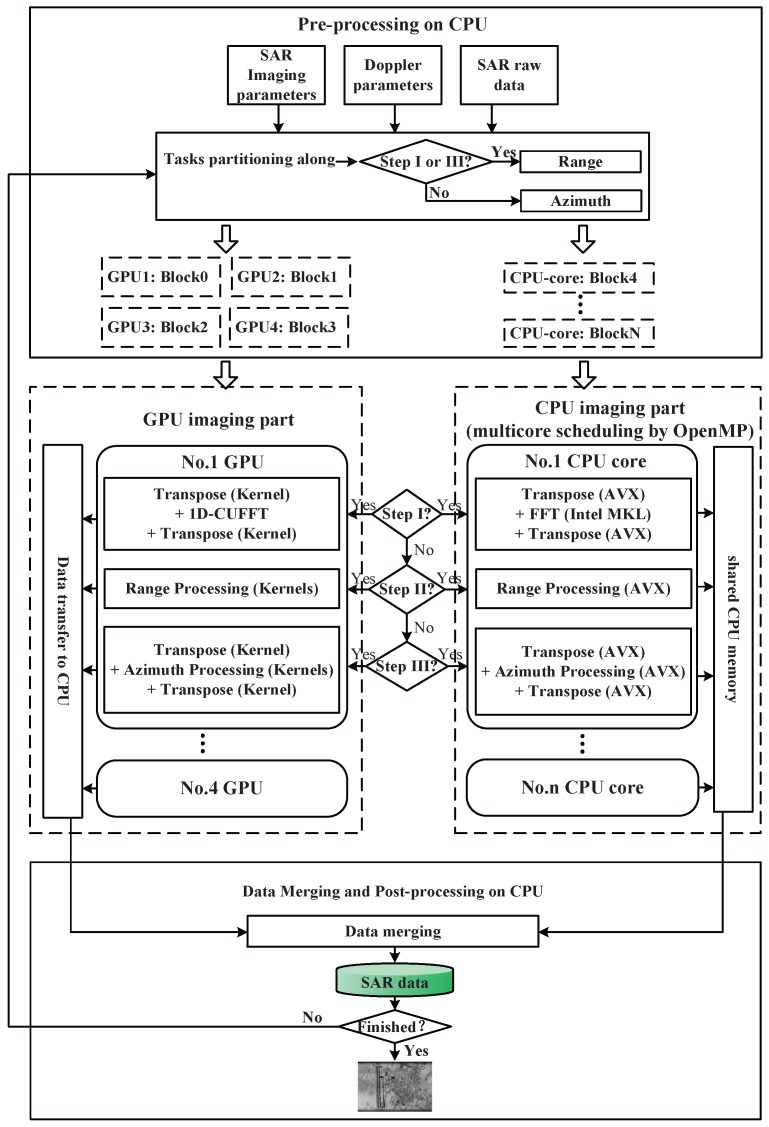
Multiple CPU/GPU deep collaborative imaging framework.

Third, on the basis of bi-direction partitioning and CPU multilevel parallel imaging, the multiple CPU/GPU deep collaborative imaging method is presented for realtime SAR imaging. A flowchart of the entire algorithm on the heterogeneous framework is illustrated in Figure 5. The deep collaborative imaging algorithm mainly includes the following three parts:

1. Task partitioning and scheduling. SAR raw data is divided into several parts according to the direction to be processed, and is distributed to multiple CPU/GPU. This step occurs three times during the entire imaging process, as shown in Figure 4.
2. Multilevel parallel processing algorithms. The multi-core parallel, vectorization and GPU massively parallel designs of CS critical processing steps, namely transpose, FFT and phase multiplication, and will be specifically discussed in the following subsections.
3. Data Merging. The final SAR image will be obtained after merging all the partial imaging results from different computing devices.

### 3.1. Collaborative Computing Oriented Imaging Task Partitioning and Scheduling

At first, multi-GPU should be seen on the same level with multi-core CPU. A coarse-grained partitioning is made for the SAR data, which can be processed respectively by multi-GPU and multi-core CPU.

**Figure 6:**
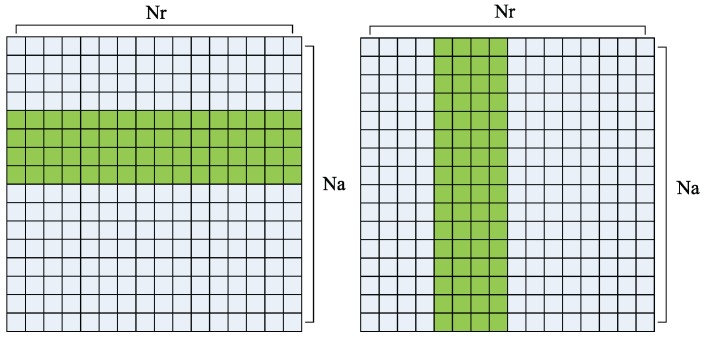
Data distribution in range (**Left**) and azimuth (**Right**).

Supposing that there are Nr points in range, which is the number of sampling points, and Na points in azimuth, which is the number of pulses. In SAR imaging processing system, SAR raw data is stored by range first. Thus I/O operations are at a high efficiency when range processing for the piece of data is stored in a segment of contiguous memory, as shown in Figure 6 (left). However, when azimuth processing, each piece of data is distributed in Na data segments, which is showed in Figure 6 (right). Each point in azimuth cannot be accessed contiguously, thus, a piece of discontinuous memory should be copied from CPU memory to GPU memory before azimuth operations. CUDA provides a function to copy a piece of discontinuous memory between CPU and GPU, *i.e.*, cudaMemcpy2D() [38].

Furthermore, using page-locked memory on CPU can improve the copy efficiency. CUDA APIs cudaHostAlloc() and cudaFreeHost() can be used to allocate and free the page-locked CPU memory [38]. Bandwidth of memory copy between GPU memory and page-locked CPU memory can be higher [38,39]. Another important reason why choosing page-locked memory is due to that copies between GPU memory and page-locked CPU memory can be performed concurrently with kernel execution on some devices of compute capability 1.1 and higher [39]. Some devices of compute capability 2.x and higher can perform a copy from page-locked host memory to GPU memory concurrently with a copy from GPU memory to page-locked CPU memory [39].

**Figure 7:**
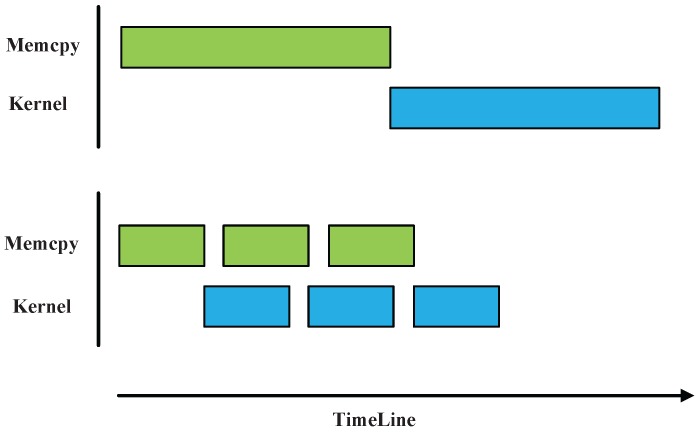
Synchronous and asynchronous execution execution.

Secondly, there is a fine-grained partitioning of data on each GPU card to realize asynchronous concurrent execution. The principle of asynchronous concurrent execution is illustrated in Figure 7. With partitioning of data and stream operations, memory copy and kernel execution, and memory copies of different directions that belong to different streams can be executed concurrently. Note that memory allocated in CPU must be page-locked and the memory copy functions are suffixed by Async like cudaMemcpy2DAsync() or cudaMemcpyAsync() when expecting an asynchronous concurrent execution [39].

In the method, 3 streams are created to concurrently execute different task. Then, data distributed to each GPU card is divided into 32 blocks, which is determined through the analysis of GPU memory and data volume. The first 3 blocks of data with stream0, stream1 and stream2, and the rest of data blocks can reuse stream0 to stream2. In this case, for the overall task, different type of tasks are assigned to different streams under overlapping execution, and expect for 3 times acceleration.

### 3.2. Transpose Optimization

In general, SAR data is stored by range-first and transposed when operating in the azimuth. At present, the main methods to achieve matrix transpose include in row out column (IROC) or in column out row (ICOR); two-page or three-page transpose method and so on [40]. CUDA 6.5 provides sample program about matrix transposing, which can realize optimization of transposing on GPU. This sample is applied into the proposal directly.

**Figure 8:**
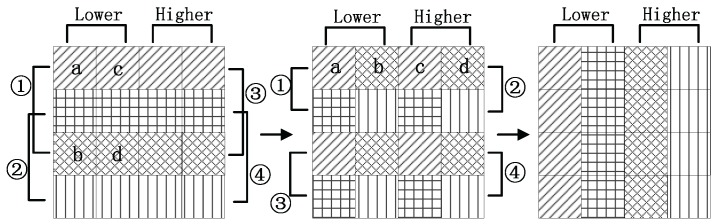
Transpose SAR data by interleaving instructions.

As for CPU, there is a new method designed to transpose the matrix with vector extension model. As shown in Figure 8, SAR data is split into blocks. Each block is composed by 4×4 pieces of 32-bits single precision floating point data, which means there are 4 rows in each block, and 4 pieces of single precision floating point data in each row. One block at range is selected each time and processed according to the next three steps:

1. Load each row of this block into a 128 bits register defined by __m128.
2. Interleave the lower (or higher) two pieces of data, which occupy 0–63 bits in the register of 128 bits, with the lower (or higher) two pieces of data using function _mm_unpacklo_ps(). Through this step, the data named a, b, c and d shown in the top data cell is rearranged to the middle data cell in a new order.
3. Store them into a temporary register.

In the first stage, the data is chosen in a certain order:

1. Lower data in 1st and 3r drows.
2. Lower data in 2nd and 4th rows.
3. Higher data in 1st and 3rd rows.
4. Higher data in 2nd and 4th rows.

So far, data cells in the middle are obtained. In the next stage, the similar process is repeated on the temporary register. Since the sequence changes, the data is modified in a new order:

1. Lower data in the 1st and 2nd rows.
2. Higher data in the 1st and 2nd rows.
3. Lower data in the 3rd and 4th rows.
4. Higher data in the 3rd and 4th rows.

The operations on the data cells are all done in an order of ➀, ➁, ➂, and ➃ as shown in figures, and when it is done, the last data cell is obtained in Figure 8, which represents the expected results. They are stored into the location that is mapped symmetrical by the diagonal with the original location. This procedure is similar to a Rubik’s cube. It is disorganized at first, and then set back in another sequence.

### 3.3. FFT Optimization

There are 4 FFT operations during the CS imaging process. Due to the fact that FFT is a basic processing tool, there are mature FFT libraries on GPU and CPU. cuFFT library is a NVIDIA CUDA FFT product and designed to provide high performance FFT operations on GPUs [41]. After cuFFT plans being created, parameters of plan can be configured by the number of pulses and sampling points. In addition, CUDA also provides API helping cuFFT plans to be set to streams, namely cufftSetStream(). Otherwise, cuFFT can also execute concurrently with memory copying.

As for CPU, MKL is used to implement FFT. MKL includes highly vectorized and threaded FFT and some other math functions. FFT of MKL is deeply optimized. Combining with OpenMP, it can realize further optimization of FFT on CPU. Otherwise, both FFT libraries are suitable for the proposed multiple CPU/GPU based collaborative imaging framework.

### 3.4. Phase Multiplication Optimization

Except of the transpose and FFT operations, phase multiplication is the main parts of the CS algorithm and occupies the most computing time. Whether chirp scaling, range compression or azimuth compression, are all seen as the phase multiplication process, namely the vector multiplication.

**Figure 9:**
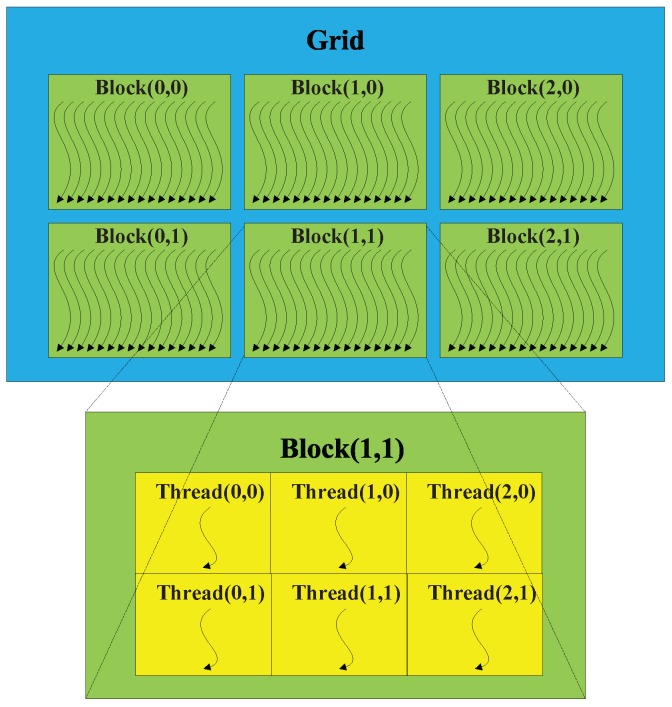
Multiple equally-shaped thread blocks.

GPU cards of NVIDIA have been developed as a high performance computing platform with high parallelism, many-cores and ultra high bandwidth, so that phase multiplication computing tasks is easy to transplant to GPU by CUDA. After designing the phase multiplication kernel, the vector multiplications can be executed by multiple thread blocks, as shown in Figure 9.

**Figure 10:**
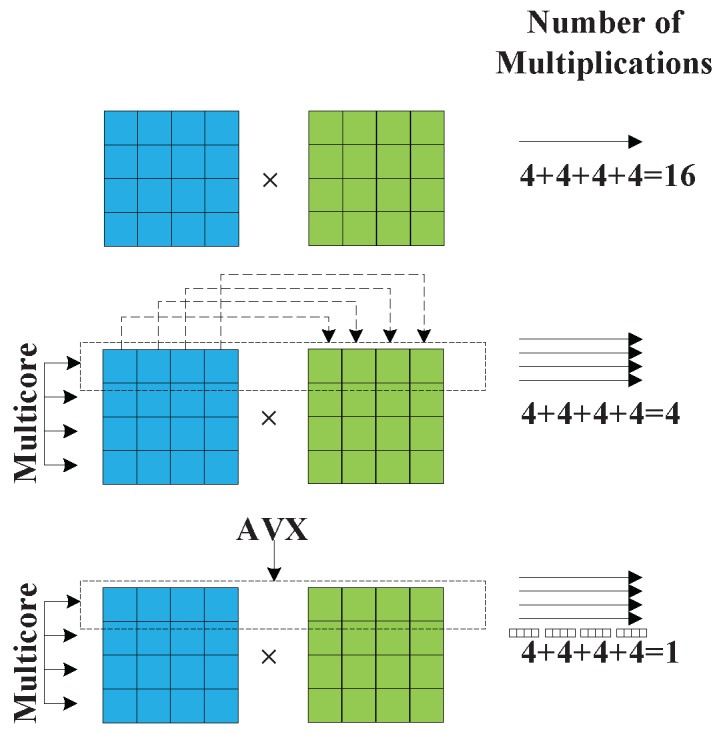
Parallel phase multiplication with OpenMP and AVX.

As for CPU implementation, the vector multiplications and the operations among the SAR raw data are not related. Therefore, it is not complex to carry out parallel computation on multi-core CPU. Since SAR data is two-dimensional (including azimuth and range), there are 2 level loops used in the program. OpenMP is used to accelerate the outside layer’s loop, where it is suitable to use OpenMP to do some coarse-grained parallelism. In the inside layer’s loop, some fine grained parallelism is implemented on SAR raw data [42]. AVX instructions are used to speed up the program in data-level. AVX helps to process 8 pieces of single precision floating point data with one instruction. Figure 10 shows phase multiplication with OpenMP in thread-level parallel and AVX in data-level parallel.

## 4. Experimental Section

The deep multiple CPU/GPU collaborative computing method proposed in this paper has made some contributions to fast SAR imaging. To realize the final acceleration effect, the experiments by 3 steps are evaluated. At first, CS algorithm is deployed into 4 GPU cards to realize massive parallel optimizations using CUDA, which enhances the processing efficiency compared with the existing CUDA based imaging method. Next, the CPU based method is designed with the combination of OpenMP based multi-core parallelism and AVX based vector extension, which is also proved a feasible solution to realize fast SAR imaging processing. At last, the above two proposals are combined to accelerate CS algorithm by parallel optimization, which can be seen as an application of heterogeneous parallel computing. All of the works are implemented with parallel optimizing based on the traditional CS algorithm. Neither any modifications nor replaced the double-precision floating point arithmetic with single-precision are done; thus, the precision of the proposal can meet the requirement of SAR imaging processing. Otherwise, for SAR imaging processing, the time consumption is only related to the number of received pulses and the number of sampling points of each pulse. So, regardless the type of targets is point target or extended scene, they are the same to the procedure of imaging processing for time consumption. In order to evaluate the efficiency, three different sizes of point target simulations are considered, *i.e.*, 8192×8192, 16384×16384 and 32768×32768. Then, the point target and extended scene simulation in 8192×8192 are performed to verify the imaging accuracy. All the SAR raw data are simulated by the multi-GPUs based time-domain SAR raw data simulator [43]. Two Intel Xeon E5 CPUs, four NVIDIA Tesla K10 GPUs are used in the experiments, whose imaging parameters and hardware specifications are listed in Table 1 and Table 2. Besides, the software environment includes three components, respectively, the OpenMP is employed for multicore processing, AVX is exploited for SIMD vector extension, and CUDA is used for multi-GPU parallel computing.

CS algorithm is chosen in the experiments. With SAR simulation data of C-band as data source and three optimizing strategies employed, the accelerating effects are analyzed according to the experimental results. There are three levels of optimizing strategies, namely multi-GPU based method, vector extension and multi-core CPU based method, and CPU/GPU heterogeneous collaborative computing method. Meanwhile, the single-core CPU running time of the CS algorithm is employed as a benchmark.

### 4.1. Performance Analysis on Multi-GPU Based Methods

The primary experiment is conducted on multi-GPU with CUDA, which includes four K10 cards. Time consumption of CS algorithm accelerated by this method and single-core CPU as well as the ratio of acceleration comparing to the reference experiment are listed in Table 3. Results in Table 3 demonstrate that the efficiency of SAR imaging has been improved by over 100, 174 and 202 times when processing SAR data whose size is 8192×8192, 16384×16384 and 32768×32768, respectively. With data size increasing, performance of this method is more prominent, which proves that the computing resource of GPU is fully utilized when processing data of big size.

### 4.2. Performance Analysis on Multi-Core Vector Extension CPU Based Method

Experiments on multi-core CPUs are conducted with vector extension instructions. Twelve threads are created on CPU to realize the coarse-grained parallelism of CS algorithm by using OpenMP. Furthermore, the fine-grained parallelism is realized with AVX on each CPU core. Time consumption and ratio of acceleration of this method are listed in Table 4. It speeds up the traditional algorithm by more than 24, 33 and 38 times when processing the three groups of SAR data in different size. Similarly, the optimizing effect is more prominent with bigger data size.

### 4.3. Performance Analysis on CPU/GPU Heterogeneous Collaborative Computing Method

According to above experimental results, multi-core vector extension acceleration performs nearly as well as CUDA method. Experiments are further designed to take the computing resources of both CPU and GPU cards into account, namely the deep heterogeneous CPU/GPU collaborative computing strategy. The experimental results are listed in Table 5, which indicates the algorithm is speeded up by more than 137, 212 and 277 times corresponding to the three groups of SAR data in different sizes, and the CPU/GPU heterogeneous collaborative computing method is affirmed to be a better strategy.

**Figure 11:**
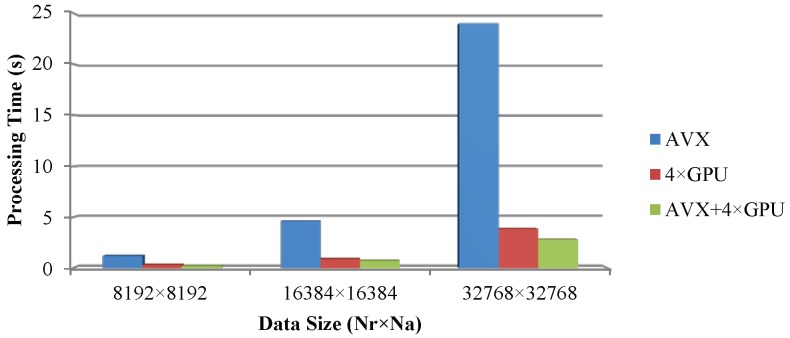
The performance comparison among three HPC methods.

There is a comprehensive comparison about the three high performance computing methods based experiments, which is illustrated in Figure 11. The conclusion is obvious that this deep CPU/GPU heterogeneous collaborative computing proposal makes the traditional CS algorithm far more efficient.

### 4.4. Accuracy Analysis on CPU/GPU Heterogeneous Collaborative Computing Method

**Figure 12:**
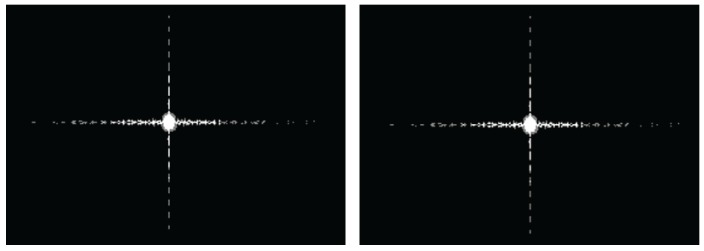
The imaging results from serial imaging (**Left**) and CPU/GPU collaborative computing (**Right**).

**Figure 13:**
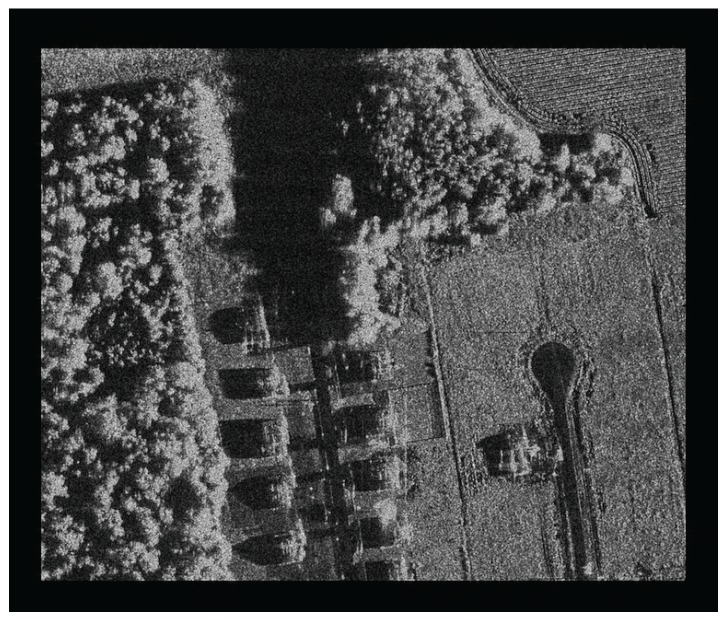
The imaging result of distributed target based on proposed method.

Figure 12 illustrates the imaging results, whose size is 8192×8192. The left one represents the result based on the single-core CPU baseline without optimization, and the right one represents the result of proposed CPU/GPU collaborative method. They are pretty much indistinguishable. Table 6 shows the indicators of imaging result in azimuth direction. Table 7 shows the indicators in range direction, where *R* represents the resolution, *E* represents the Expansion ratio, *P* represents the peak sidelobe ratio, and *I* represents the integral sidelobe ratio. There are no differences between them. Furthermore, a simulated raw data of extended scene is processed by the proposed collaborative computing method, and its result is shown in Figure 13. Here is just focusing capability verification, one SAR image is taken as the scene reflectivity map without considering the scene geometric and electromagnetic model. The future work will be the classical extended scene simulation [4,5,6,7,8,9,10] for the geometric and radiometric accuracy verification of imaging algorithm.

Through the above accuracy analysis on imaging indicators and image result, the accuracy of the proposed collaborative computing method can be totally guaranteed. There is another implementation related issue should be additionally considered, namely the data type of the imaging code. For the CS imaging, the single-precision floating point type (float) is accurate enough for indicator analysis, target recognition and other SAR image applications, and is also employed in the imaging program. However, for the further interferometric processing, the preserved phase information by float operation may not accurate enough. So, the data type in should be changed to double-precision floating point type (double) for fully phase preserving in interferometric applications. Accordingly, the changes of data type from float to double may reduce the imaging efficiency to some extent in that the float computing power of CPU and GPU are always higher than their double computing power.

### 4.5. Cost Analysis

From the price standpoint, market prices of K10 card (with 2 GK104 cores) is about $3400, Intel Xeon E5 CPU is sold with a price about $726. All of the computing devices have taken about $7526, since SAR imaging processing has been speeded up by 270+ times when processing data of big size, the final heterogeneous collaborative computing method has a real price advantage on a multiple of 27. From the power consumption perspective, K10 consumes about 250w when computing, and the nominal value of Intel Xeon E5 CPU is 80w, which indicates a power advantage on a multiple of 37. For a portable purpose, 7 CPUs are needed to achieve the same efficiency of SAR imaging processing. Thus, it can be seen that the final CPU/GPU collaborative computing method proposed in this work has a great advantage not only from the price standpoint, but also the power consumption and portable perspectives.

## 5. Conclusions

In this paper, a deep CPU/GPU collaborative computing method was proposed to realize the deep optimization on traditional CS algorithm without modifying the algorithm itself. At first, the structure of CS algorithm was analyzed, and two optimizing strategies were presented based on multi-core GPU and vector extension based multi-core CPU respectively, which fit the structure of CS algorithm. After that, the technical challenges of the two methods were explained in details. Based on the works above, a new framework was achieved, which fused CPU and GPU together, namely the deep CPU/GPU collaborative computing method on SAR imaging processing. In the proposed method, computing resources of devices were fully utilized, not only GPU, CPU also took on a part of computing tasks. As for GPU, simulation data was partitioned to make memory copy operations and kernel functions of each block of data executed concurrently with asynchronous concurrent APIs of CUDA, which overlapped most of the memory copy operations. Beyond that, some samples and library functions provided by CUDA were helpful to enhance the efficiency of optimization. For CPU, OpenMP was employed for multi-core processing, and AVX was exploited for SIMD vector extension on each CPU core, which also enhanced the computing efficiency of CPU. In the final proposal, the two optimizing strategies were combined to further enhance the efficiency of CS algorithm. The experimental results demonstrated that this deep CPU/GPU collaborative computing method can achieve more than 270× speedup over traditional single-core CPU method when processing data of big size. In addition, it was proved more advantageous when the size of simulation data increasing.

Generally, the proposed method has proven to be highly efficient, and can be used in realtime SAR imaging processing system. Furthermore, the inverse process of the proposed method can be employed to realize the realtime inverse CS algorithm [44], which can be used to generate simulated SAR raw data. Thus, the realtime raw data generation will be implemented under the multiple CPU/GPU collaborative computing framework. The future work of this research will apply the multiple CPU/GPU collaborative computing method to SAR target detection and recognition domain [45]. 

## Figures and Tables

**Table 1:** Imaging Parameters.

Parameters	Value
Wave length	0.056 m
PRF	1850 Hz
Pulse width	30 us
Band width	60 MHz
Sampling rate	80 MHz
Center distance	1000 km

**Table 2:** Environment Specifications.

Parameters	Value
Operating system	Redhat 6.5
CPU	Intel Xeon E5-2630 v2
Number of CPU cores	12 × 2
GPU	NVIDIA Tesla K10
Number of CUDA cores	1536 × 4
GPU Float performance	1.5TFlops × 4
Total dedicated GPU memory	4GB × 4

**Table 3:** Running time of multi-GPU based method.

Data Size	4 GPU (s)	Sing-Core CPU (s)	Ratio
8192×8192	0.30	30.21	100.70
16,384×16,384	0.88	153.19	174.08
32,768×32,768	3.85	778.11	202.11

**Table 4:** Running time of multi-core vector extension CPU based method.

Data Size	SIMD CPU (s)	Sing-core CPU (s)	Ratio
8192×8192	1.21	30.21	24.97
16,384×16,384	4.61	153.19	33.23
32,768×32,768	20.33	778.11	38.27

**Table 5:** Running time of collaborative computing based method.

Data Size	CPU/GPU (s)	Sing-Core CPU (s)	Ratio
8192×8192	0.22	30.21	137.32
16,384×16,384	0.72	153.19	212.76
32,768×32,768	2.8	778.11	277.90

**Table 6:** Imaging indicators of azimuth direction.

Scene Size	Baseline	CPU/GPU Based Method	Error
R (m)	3.76	3.76	0.00
E (dB)	0.82	0.82	0.00
P (dB)	−12.99	−12.99	0.00
I (dB)	−9.6	−9.6	0.00

**Table 7:** Imaging indicators of range direction.

Scene Size	Baseline	CPU/GPU Based Method	Error
R (m)	3.19	3.19	0.00
E (dB)	0.88	0.88	0.00
P (dB)	−13.31	−13.31	0.00
I (dB)	−9.95	−9.95	0.00

## Abbreviations

The following abbreviations are used in this manuscript:

SAR: Synthetic aperture radar
GPU: Graphics processing unit
CS: Chirp scaling
SIMD: Single instruction multiple data
AVX: Advanced vector extensions
